# Supplementary material for: Infection by Salmonella enterica Serovar Typhimurium DT104 Modulates Immune Responses, the Metabolome, and the Function of the Enteric Microbiota in Neonatal Broiler Chickens
Source: Pathogens. 2022 Oct 29;11(11):1257. doi: 10.3390/pathogens11111257 (PMC9694942; doi:10.3390/pathogens11111257)
Supplement: Supplementary file 1 [file pathogens-11-01257-s001.zip › pathogens-1945835-supplementary.pdf]

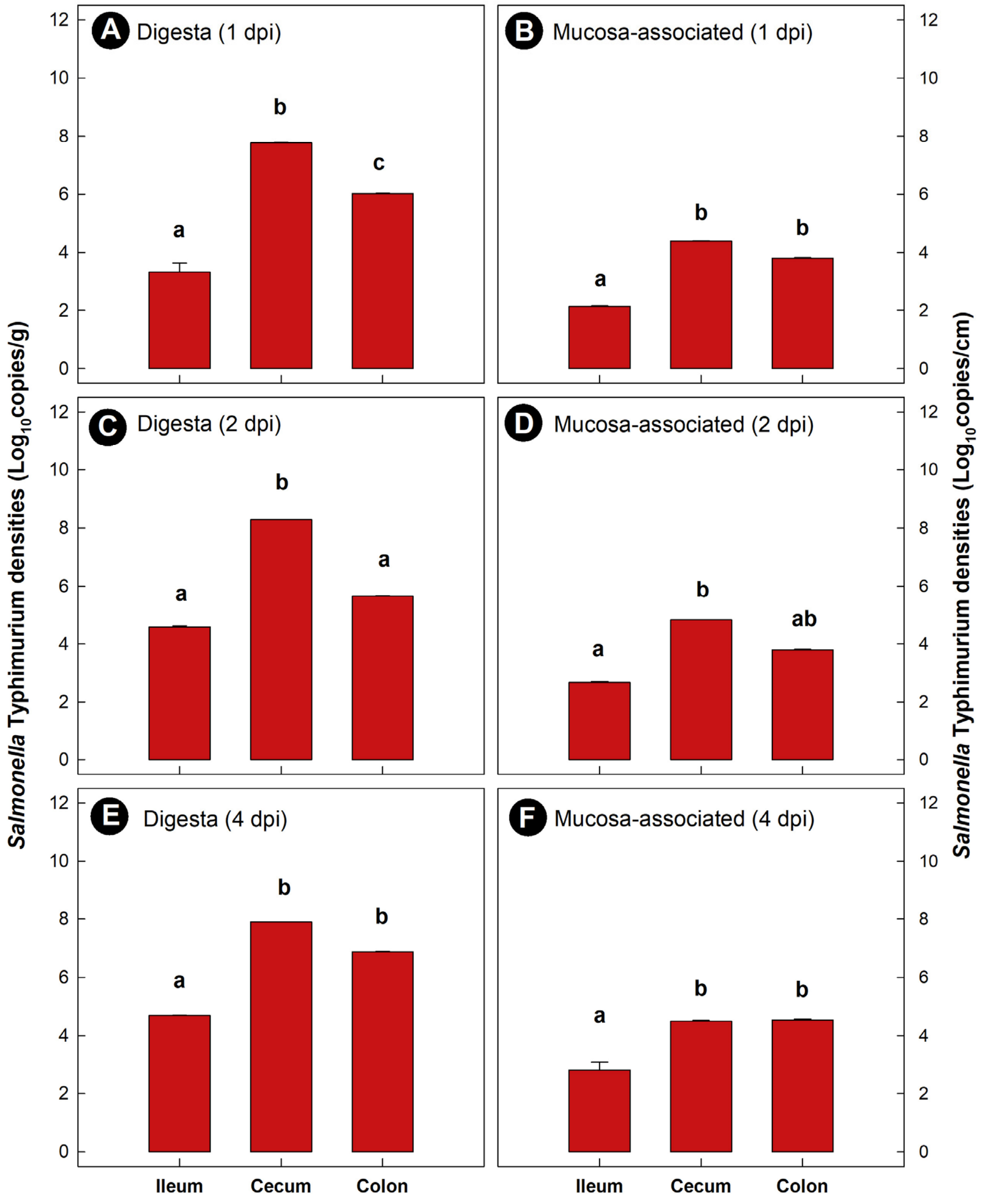

Figure S1. *Salmonella enterica* serovar Typhimurium densities from digesta and associated with mucosa from the ileum, cecum, and colon of infected broiler chicks at 1, 2 and 4 days post-inoculation (dpi). (**A**) Digesta at 1 dpi. (**B**) Mucosa-associated at 1 dpi. (**C**) Digesta at 2 dpi. (**D**) Mucosa-associated at 2 dpi. (**E**) Digesta at 4 dpi. (**F**) Mucosa associated at 4 dpi. Vertical lines associated with histogram bars represent standard errors of the means (n = 6). Histogram bars not denoted with the same letter differ ($p \leq 0.050$).

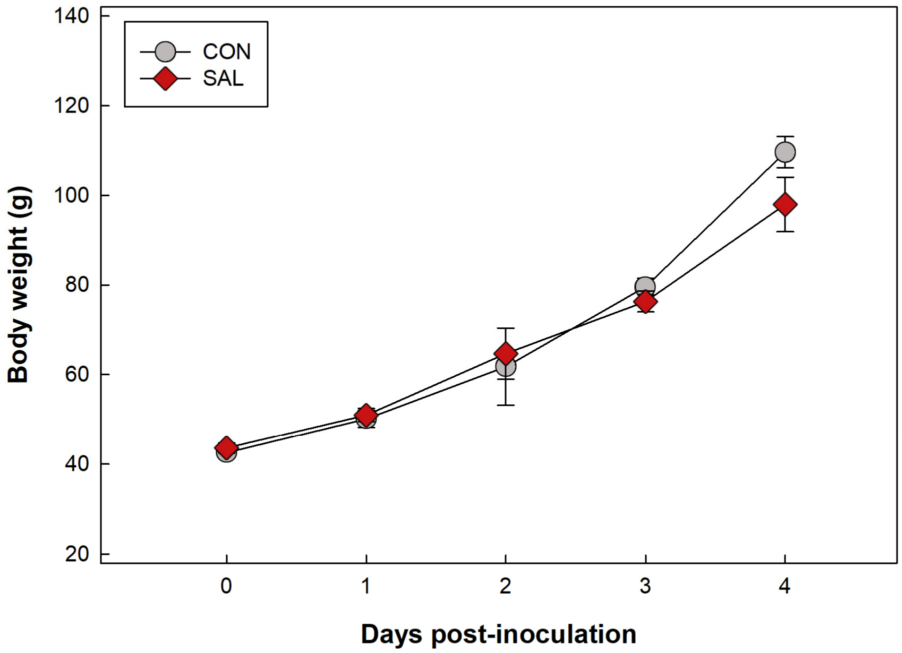

Figure S2. Temporal changes in body weight of chicks inoculated with *Salmonella enterica* serovar Typhimurium (SAL) or administered medium alone (CON). Vertical lines associated with markers represent standard errors of the mean (n ≥ 6).

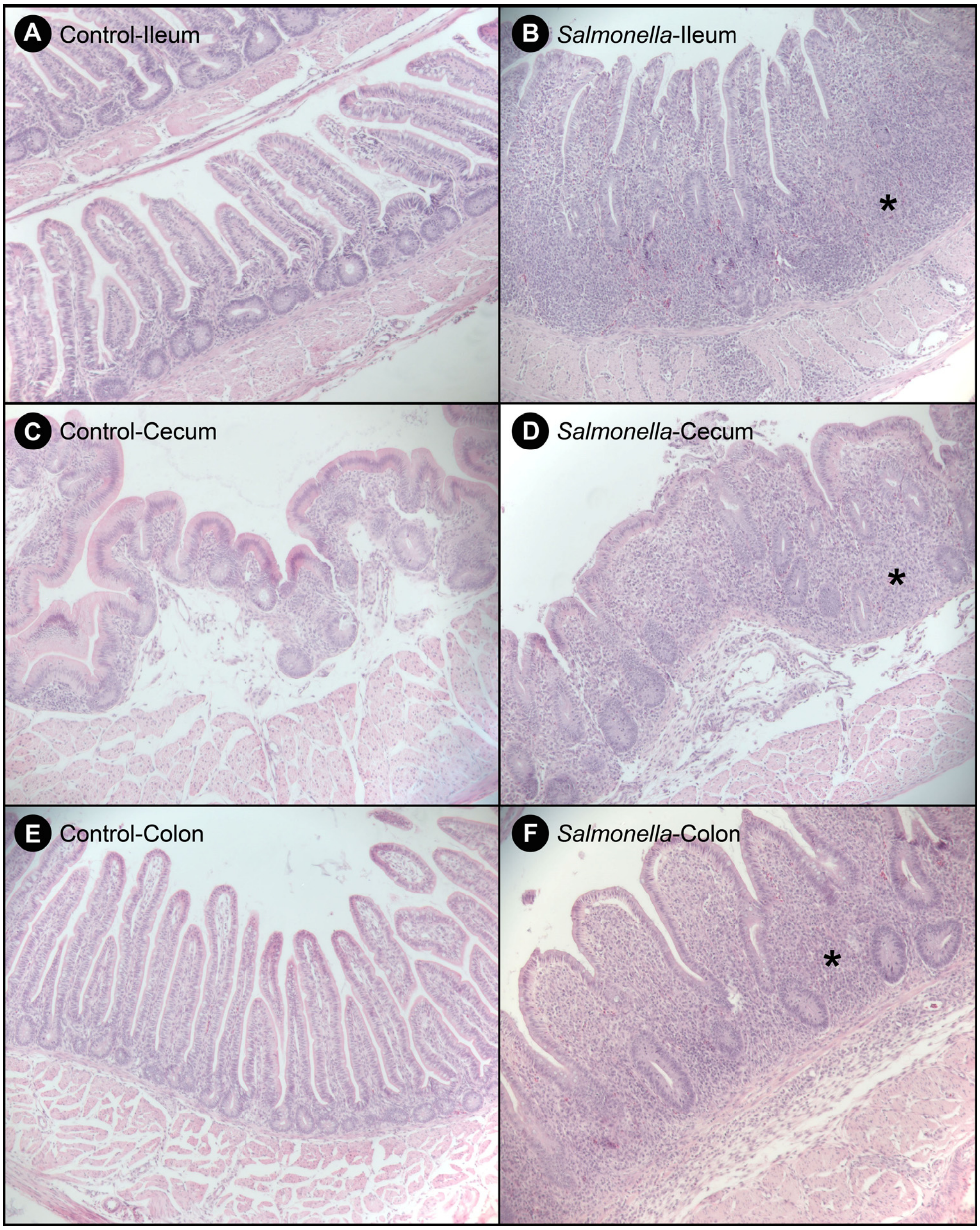

Figure S3. Histological representation of intestinal tissues from broiler chicks inoculated with *Salmonella enterica* serovar Typhimurium (*Salmonella*) or administered medium alone (Control) at 4 days post-inoculation. Asterisks indicate infiltration of immune cells. (**A**) Control-ileum. (**B**) *Salmonella*-ileum. (**C**) Control-cecum. (**D**) *Salmonella*-cecum. (**E**) Control-colon. (**F**) *Salmonella*-colon.

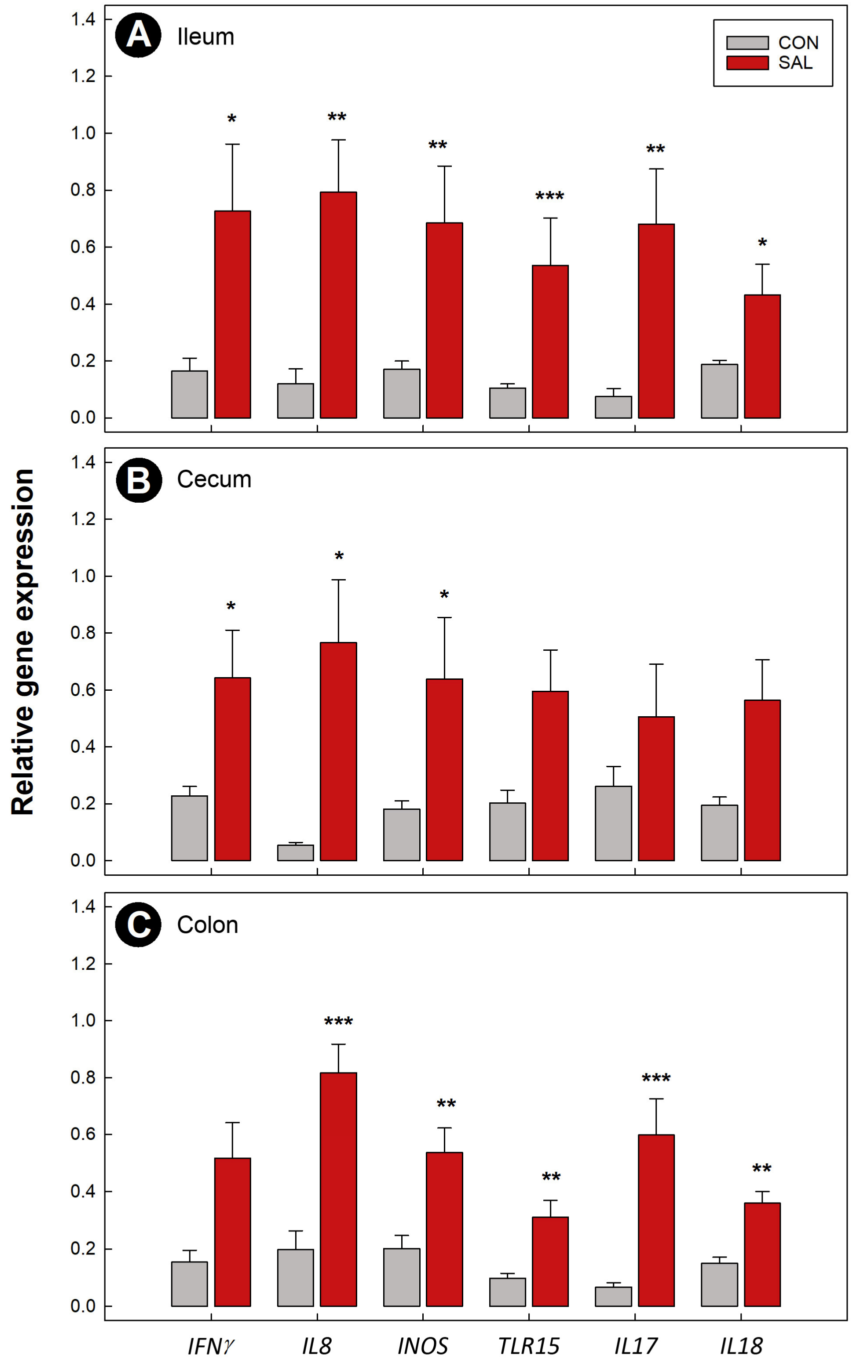

Figure S4. Relative expression of immune genes in broiler chicks inoculated with *Salmonella enterica* serovar Typhimurium (SAL) or administered medium alone (CON) at 2 days post-inoculation. (**A**) Ileum. (**B**) Cecum. (**C**) Colon. Vertical lines associated with markers are standard errors of the mean (n = 6). Histogram bars with asterisks indicate a difference (**p* ≤ 0.050, ***p* ≤ 0.010, ****p* ≤ 0.001) between the CON and SAL treatments.

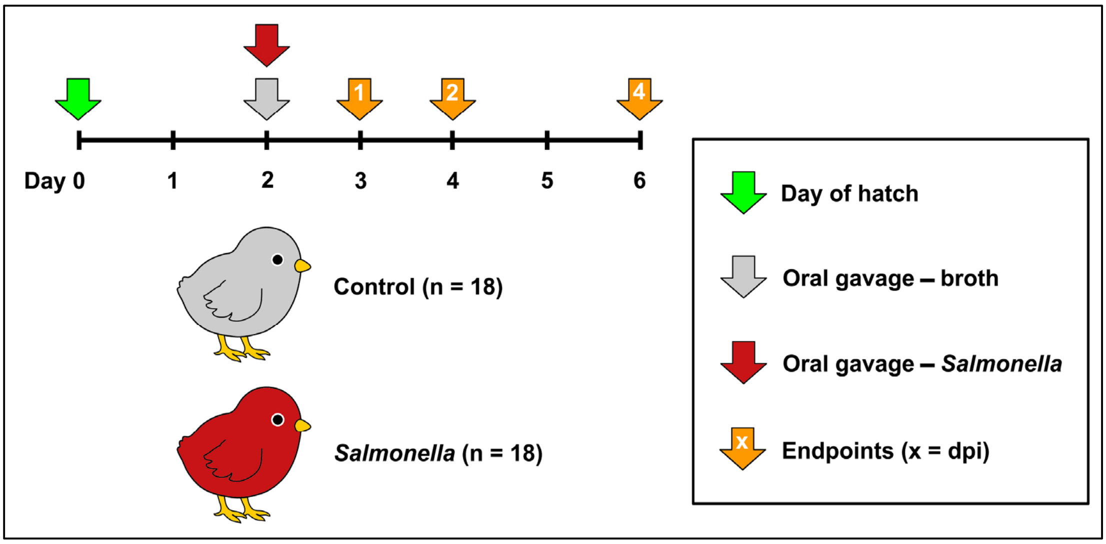

Figure S5. Treatments and the experimental timeline. The experiment was arranged as a factorial experiment with two levels of treatment (Control and *Salmonella enterica* serovar Typhimurium) and three levels of time (1, 2, and 4 days post-inoculation [dpi]). Each treatment-time point consisted of six replicate chicks (36 total).

Table S1. Number of metabolite bins that differed between broiler chicks inoculated with *Salmonella enterica* serovar Typhimurium (or administered medium alone (control treatment) by sample type and days post-inoculation (dpi).

**Sample type**	**Number of metabolite bins**		
	**1 dpi**	**2 dpi**	**4 dpi**
Ileum digesta	-	8	36
Breast	8	20	66
Liver	21	11	29
Serum	21	12	19
Hippocampus	5	13	6

Table S2. List of primers used to quantify mRNA concentrations of immune and house-keeper genes.

Target gene	Primer		Sequences (5' to 3')	T_a (°C)[1]	Source
β-Actin	*β-ACT*	Fwd	CTCTGACTGACCGCGTTACT	58	PMID[2]: 31848364
		Rev	TACCAACCATCACACCCTGAT		
Glyceraldehyde-3-phosphate dehydrogenase	*GADPH*	Fwd	GGTGGTGCTAAGCGTGTTAT	58	PMID: 26908883
		Rev	ACCTCTGTCATCTCTCCACA		
Inducible nitric oxide synthase	*INOS*	Fwd	GAACAGCCAGCTCATCCGATA	54	PMID: 17709416
		Rev	CCCAAGCTCAATGCACAACTT		
Interferon gamma	*IFNγ*	Fwd	GCCGCACATCAAACACATATCT	57	PMID: 17709416
		Rev	TGAGACTGGCTCCTTTTCCTT		
Interleukin 8	*IL8*	Fwd	ATGAACGGCAAGCTTGGAGCT	57	PMID: 17709416
		Rev	GCCATAAGTGCCTTTACGATCAG		
Interleukin 10	*IL10*	Fwd	CGCTGTCACCGCTTCTTCA	57	PMID: 25653004
		Rev	TCCCGTTCTCATCCATCTTCTC		
Interleukin 17	*IL17*	Fwd	AGATGCTGGATGCCTAACCC	56	PMID: 25935756
		Rev	GTGGTCCTCATCGATCCTGTAA		
Interleukin 18	*IL18*	Fwd	GTGAAGAGATCGCTGTGTGT	56	PMID: 12902481
		Rev	ATCGCATTCCAGCTCATCATC		
CCL4 (MIP-1β)	*MIP1β*	Fwd	GTGTTGTGTTCATCACCAGGAA	56	PMID: 16782198
		Rev	GCAAAGTGGGCATAGCGTG		
Toll-like receptor 4	*TLR4*	Fwd	TCTTTCAAGGTGCCACATCC	56	PMID: 30532755
		Rev	TTTCAGCGACGTTAAGCCAT		
Toll-like receptor 15	*TLR15*	Fwd	ACTAGAACCTGCCTGCCAAC	56	PMID: 23352867
		Rev	AGCATCAGGAGAGATTGCCC		
Transforming growth factor β2	*TGFβ2*	Fwd	CCATCTACAACAGCACCAGGG	58	PMID:
		Rev	TAGCTTGGTGGGATGGCATTTTC		24299181

[1]Annealing temperature.
[2]PubMed unique identifier.
